# The mental health effects of critical illness insurance: Evidence from China’s aging population

**DOI:** 10.1371/journal.pone.0333546

**Published:** 2025-11-06

**Authors:** Yuehong Zhang, Manxia Tian, Yumiao Zhang, Wenbin Zang

**Affiliations:** 1 School of Public Administration, Gansu University of Political Science and Law, Lanzhou, Gansu, China; 2 Hebei Academy of Social Sciences, Shijiazhuang, Hebei, China; 3 School of Economics, Lanzhou University of Finance and Economics, Lanzhou, Gansu, China; 4 School of Public Administration. Southwestern University of Finance and Economics, Chendu, Sichuan, China; Mansoura University Faculty of Veterinary Medicine, EGYPT

## Abstract

**Background:**

With the rapid progression of aging, mental health challenges among the elderly have become increasingly pronounced. Addressing these issues is vital for enhancing older adults’ quality of life and maintaining social stability. This study investigates the impact of China’s Critical Illness Insurance (CII) policy, a pivotal component of the national healthcare system, on the mental health of older adults.

**Methods:**

This study leverages data from the China Health and Retirement Longitudinal Study (CHARLS) and utilizes the staggered implementation of the CII policy across regions as a “quasi-natural experiment.” A multi-period difference-in-differences (DID) approach is employed to estimate the policy’s effects. The analysis focuses on depression tendencies and scores among older adults, controlling for key sociodemographic and economic covariates.

**Results:**

The findings reveal three key insights: The CII policy significantly reduced depressive tendencies and depression scores among older adults, demonstrating marked improvements in their mental health.The policy effects exhibit substantial heterogeneity, with more pronounced benefits observed among middle- and high-income groups, individuals with chronic illnesses, and older adults in central and western regions.Mechanism analysis indicates that the policy alleviates psychological distress and enhances household financial stability by increasing access to healthcare services, sharing medical expenses, and mitigating financial risks.

**Conclusion:**

The CII policy has proven effective in improving the mental health of older adults, with significant variations across income levels, health conditions, and regional economic development. By reducing financial stress and improving access to healthcare, the policy not only addresses mental health disparities but also bolsters household economic resilience. These findings underscore the critical importance of tailored health insurance policies to address the diverse needs of an aging population effectively.

## 1. Introduction

As the global elderly population continues to expand, the world is entering what is termed The Age of Longevity. According to data from the United Nations Department of Economic and Social Affairs Population Division, the global population aged 60 and above reached 1 billion in 2020. This figure is projected to rise to 1.4 billion by 2030, accounting for one-sixth of the global population, and to double by 2050, reaching 2.1 billion. The advent of this era of rapid aging presents significant challenges, particularly concerning the deterioration of mental health among the elderly. According to the World Health Organization, approximately 15.96 million individuals aged 60 and above globally are affected by depression [1]. Similarly, the China Health and Aging Report indicates that 33.1% of surveyed elderly individuals exhibit severe symptoms of depression [2]. The health status of the elderly population is increasingly compromised by physiological changes, shifts in socioeconomic status, and evolving social roles. Among these, mental health issues such as depression and anxiety are particularly prevalent [3]. Alarmingly, the suicide rate among older adults is significantly higher compared to other demographic groups [4].

The repercussions of mental health disorders extend beyond individual health deterioration to impose substantial financial burdens on families. The WHO reports that one in six disabilities worldwide is attributable to mental disorders. Individuals with severe mental health conditions face a reduced life expectancy, living 10–20 years less than the general population. Furthermore, mental health disorders exacerbate the risks of suicide and violations of human rights [1]. From an economic perspective, the burden of mental health issues is equally daunting. A 2018 report by Psychiatric Times estimates that by 2030, the global economic cost of mental illness will amount to $16 trillion. This staggering cost arises largely from the early onset of mental disorders and the associated loss of productivity, with an estimated 12 billion workdays lost annually due to mental illnesses. Although mental and behavioral disorders contribute 12% of the global disease burden, the allocation of mental health budgets remains disproportionately low. Most countries allocate less than 1% of their total health expenditures to mental health, exposing a significant gap between disease burden and financial resources. Additionally, over 40% of countries lack dedicated mental health policies, while more than 30% have not implemented any mental health programs.

The Critical Illness Insurance (CII) policy is a key component of China’s healthcare system. Its core objective is to reduce the financial burden of medical expenses and mitigate the risk of poverty or a return to poverty caused by illness. Among the elderly population, the high prevalence of major illnesses and chronic diseases significantly increases the incidence of catastrophic healthcare expenditures at the household level [5]. By providing secondary compensation for medical expenses, CII not only delivers direct financial support but also alleviates the budgetary strain caused by medical expenditures, thereby freeing up household financial resources to meet other non-medical needs. This reallocation of resources reduces economic pressure and enhances individuals’ sense of financial security, which in turn improves mental health [6]. For low-income groups, the economic shock resulting from high medical expenses constitutes a major source of psychological stress. According to stress mitigation theory, reducing financial stressors can significantly enhance an individual’s mental health. By lowering the risk of poverty due to illness and improving the accessibility of healthcare resources, CII plays a vital role in improving the mental health of the elderly.

Research on the relationship between health insurance and health has primarily focused on the impact of health insurance schemes for urban residents or employees, particularly in terms of promoting physical health among insured populations, including rural residents and other vulnerable groups. However, studies on the relationship between Critical Illness Insurance (CII) policies and health have predominantly concentrated on their role in improving physical health and alleviating economic burdens, with relatively little attention paid to their effects on mental health. This paper aims to fill this gap by specifically examining the impact of the major illness insurance policy on the mental health of older adults. It employs a systematic empirical causal analysis and path analysis, along with a multi-dimensional heterogeneity analysis. Through these analyses, the paper provides policy recommendations for optimizing health insurance policies and improving the mental health of the elderly.

Using data from the China Health and Retirement Longitudinal Study (CHARLS), this study explores the mechanisms and specific impacts of CII policies on the mental health of older adults.The findings reveal that CII policies significantly reduce depression scores and the prevalence of depressive tendencies among the elderly, demonstrating their efficacy in improving mental health. Mechanistic analysis identifies four pathways through which CII positively influences mental health: medical service utilization, medical cost-sharing, financial risk prevention and control, and social support. Specifically, increased inpatient service utilization emerges as the core driver of the policy’s impact on mental health. By enhancing reimbursement rates for hospitalization expenses, the policy alleviates the financial pressure associated with hospitalization and reduces the risk of poverty or financial relapse caused by illness. Moreover, the policy effectively decreases the probability of catastrophic health expenditures, playing a pivotal role in financial risk prevention and control.Within the social support framework, economic and social support positively contribute to improved mental health, while emotional support demonstrates some negative effects. Further heterogeneity analysis reveals that CII policies have a more pronounced impact on the mental health of middle- and high-income groups, individuals with chronic illnesses, and elderly populations in central and western regions. These findings indicate that the policy’s effects vary across populations with different income levels, health statuses, and regional economic development levels.Amid the intensifying trend of population aging, this study provides policymakers with empirically grounded insights to support the continuous improvement of elderly healthcare services within China’s social healthcare insurance system. These findings serve as a valuable reference for enhancing the quality of life of the elderly.

## 2. Institutional background and literature review

### 2.1. Institutional background

The Critical Illness Insurance (CII) for urban and rural residents is a policy arrangement built upon basic medical insurance to provide additional protection for patients facing high medical costs. Its purpose is to expand the coverage of basic medical insurance and improve its protective effectiveness. As an extension and supplement to basic medical insurance, CII alleviates patients’ financial burdens through additional compensation for high medical expenses, enhances the accessibility of healthcare services, and significantly improves social welfare. The implementation of this policy has strengthened the framework of the healthcare insurance system and provided crucial support for addressing the issues of poverty and financial relapse caused by illness.

Since the policy’s launch in August 2012, the development of the CII system for urban and rural residents in China has undergone several stages. Initially, during the pilot phase, on August 24, 2012, six ministries, including the National Development and Reform Commission, jointly issued the Guiding Opinions on Conducting Critical Illness Insurance for Urban and Rural Residents, which proposed establishing a CII system covering urban and rural residents. The policy required local governments to implement pilot projects step by step, with a focus on defining reasonable compensation mechanisms and ensuring coverage for rural residents and non-working urban residents. The comprehensive implementation phase began on July 22, 2015, when the State Council executive meeting officially mandated the nationwide rollout of CII. In August of the same year, the government issued the Opinions on Fully Implementing Critical Illness Insurance for Urban and Rural Residents, marking the system’s formal nationwide implementation.

In February 2020, the Chinese government clarified its goal of strengthening the “triple-layered protection system,” which integrates basic medical insurance, critical illness insurance, and medical assistance to better address severe diseases and diverse medical needs. In May 2021, the Administrative Measures for the Critical Illness Insurance Business of Insurance Companies were introduced, aiming to establish a regulatory framework covering all aspects and stages of CII. Through continuous policy optimization, the CII system has been progressively refined and integrated with other medical insurance schemes, forming a complementary mechanism and laying a solid foundation for building a fairer, more efficient, and multi-tiered healthcare security system.

### 2.2. Literature review

Existing research widely supports the positive role of health insurance in improving residents’ health. Studies have shown that the enrollment rate in health insurance significantly increases the utilization of medical services, thereby demonstrating a significant positive correlation with improvements in residents’ self-rated health, a conclusion that has been verified in multiple studies [7–9]. Individuals without public or private health insurance are at higher risk of all-cause and cancer-related mortality [10]. In the context of China, enrollment in basic health insurance has effectively alleviated the economic burden of insured individuals, significantly improving the overall health level of rural residents, and playing a crucial role in reducing household financial risks and alleviating poverty [11, 12]. Moreover, compared to rural health insurance, urban residents’ health insurance has been shown to have a significant health-promoting effect on those with poorer health [13]. Research has also found that employee-based health insurance significantly increases the utilization of outpatient medical services, but its direct impact on improving health outcomes is relatively limited [14]. At the same time, the urban residents’ basic health insurance has improved the health levels of workers with poorer health, enabling them to return to the labor market and increasing employment opportunities for such individuals [15]. The urban-rural health insurance reform, by reducing out-of-pocket medical expenses, has not only improved the financial risk protection for rural residents to some extent, but also significantly enhanced their self-rated health [12]. However, some studies have questioned the actual effect of health insurance on improving health, suggesting that certain healthcare expenditures have not resulted in significant health benefits, and there is still room for improvement in the efficiency of medical resource use [16]. In related research in China, the new rural cooperative medical insurance has not led to significant improvements in the health status of insured rural residents, especially children [17].

As a vulnerable group, older adults face higher medical needs [18]. In this regard, health insurance is widely regarded as having a significant impact on the medical expenditures and health of older adults. Some studies have indicated that enrollment in basic health insurance promotes the use of medical services among older adults, improving their health and narrowing health disparities [19, 20]. Further empirical analysis has shown that the new rural cooperative medical insurance has had a particularly significant role in the prevention of specific chronic diseases, reducing the incidence of stroke and diabetes among rural residents aged 55 and above [21]. The urban residents’ basic health insurance policy also has positive implications for improving the health outcomes of middle-aged and elderly rural populations, partially narrowing the urban-rural health gap [20]. Despite these findings, there are still doubts about the effectiveness of health insurance for different groups. Research points out that employee health insurance has a significant health-promoting effect on elderly individuals with high healthcare service utilization, but the new rural cooperative medical insurance has failed to improve the health status of older adults effectively [22]. Furthermore, the health-promoting effect of health insurance on older adults shows significant differences between urban and rural areas, suggesting that the effectiveness of health insurance policies is limited by regional development levels and socio-economic conditions [23].

As an important component of China’s healthcare security system, the Critical Illness Insurance (CII) policy aims to alleviate the economic burden of patients with major illnesses by reducing their medical expenses and preventing the consumption and wealth loss caused by health shocks among older adults [24–26]. Existing research has mainly assessed the impact of this system on alleviating the economic burden of patients. Specifically, the implementation of the CII policy has significantly reduced out-of-pocket medical expenses and increased the reimbursement ratio for medical costs [27], which has, to some extent, reduced the incidence and intensity of catastrophic health expenditures [28]. Furthermore, the CII policy has had a lasting effect on improving the health levels of rural patients [29], leading to an increase in disposable income and improvements in consumption [30]. Nevertheless, some studies have found that the CII policy has not had a significant effect on reducing the incidence of catastrophic health expenditures [31], and its level of protection in alleviating economic burdens remains limited [32, 33].

A review of the existing literature reveals several key gaps. First, most research focuses on the role of health insurance in improving physical health and reducing financial burdens, with relatively little attention paid to its impact on the mental health of older adults—a crucial dimension. Few studies have systematically examined the effects of major illness insurance on elderly mental health. Second, while many studies assess the impact of major illness insurance on catastrophic health expenditures and economic burdens, there is a lack of in-depth analysis regarding its broader health effects on older adults. Discussions about how major illness insurance indirectly influences mental health through specific institutional pathways are particularly underdeveloped, limiting a comprehensive understanding of its policy efficacy. Third, the health effects of major illness insurance exhibit significant heterogeneity due to variations in regional economic development, healthcare resource allocation, and population characteristics. However, existing research rarely conducts detailed analyses based on regional or socioeconomic differences, thereby constraining theoretical support for policy optimization and targeted implementation.

## 3. Material and methods

### 3.1 Data collection

The data utilized in this study are primarily sourced from the China Health and Retirement Longitudinal Study (CHARLS), a large-scale, nationally representative longitudinal survey. CHARLS is led by the National School of Development at Peking University and executed by the university’s China Center for Social Science Survey (CCSSS). The project is dedicated to collecting high-quality data on individuals aged 45 and above, along with their households, to provide a comprehensive understanding of the middle-aged and elderly population in China.The CHARLS dataset encompasses 150 counties and 450 communities (villages) across 28 provinces, autonomous regions, and municipalities in China. The baseline survey was conducted in 2011, followed by three waves of follow-up surveys in 2013, 2015, and 2018. This temporal structure aligns well with the requirements of this study, allowing for the construction of a complete research sample using data from all four waves. It also facilitates the application of the difference-in-differences (DID) methodology to estimate the impact of the implementation of the critical illness insurance (CII) policy.The CHARLS dataset provides a rich array of individual-level information, including respondents’ basic demographic characteristics, family structure, health status, healthcare utilization, employment and income, and community environment. This extensive information serves as a critical foundation for analyzing the psychological health of the elderly and its determinants. Moreover, it underpins the exploration of the potential pathways through which the CII policy affects mental health outcomes, offering valuable insights into its broader implications.

In this study, we employ a multi-period Difference-in-Differences (DID) methodology to identify the impact of the Critical Illness Insurance (CII) policy on the mental health of the elderly. Given the varying implementation timelines of the CII policy across different regions in China, we manually compiled the policy implementation timeline for each region. Specifically, the policy was first piloted in certain cities starting in 2012, and gradually expanded to more cities thereafter. Meanwhile, the CHARLS data covers four waves of data collected in 2011, 2013, 2015, and 2018, which allows us to capture the policy’s implementation across different time points and regions. To construct the multi-period DID model, we define two key variables: treat, which indicates whether a region belongs to the treatment group (i.e., the regions that eventually implemented the policy), and post, which indicates whether the region has entered the policy implementation phase. The interaction term between these two variables represents whether an individual in a specific year and region has been exposed to the policy. We use this interaction term as the core explanatory variable to identify the net effect of the policy.

### 3.2 Model specification and variable definition

Since 2012, China has been piloting a Critical Illness Insurance (CII) system in selected regions, aiming to alleviate the high medical cost burdens faced by urban and rural residents due to major illnesses. The pilot phase received significant attention and active responses from various regions. By the end of 2013, 25 provinces and municipalities had introduced pilot implementation plans for CII, covering 134 cities and towns. This pilot phase provided a strong foundation for the nationwide rollout of the policy.

By the end of 2014, the CII system had expanded to 27 provinces and municipalities, covering hundreds of millions of urban and rural residents. In August 2015, the government issued the Opinions on Comprehensively Implementing Major Disease Insurance for Urban and Rural Residents, which explicitly mandated that by the end of 2015, the CII system should cover all urban and rural residents enrolled in basic health insurance. According to this policy document, a more comprehensive nationwide CII system was gradually established by 2017 to reduce catastrophic healthcare expenditures associated with major illnesses. As the policy continued to be rolled out and implemented across various regions, the coverage of CII steadily expanded and became institutionalized nationwide.

The variation in the timing of CII implementation across regions offers a valuable quasi-natural experiment for evaluating the policy’s effects. This allows for the use of a multi-period difference-in-differences (DID) model to assess the specific impacts of the policy. This phased implementation provides a robust background for empirical analysis, enabling the identification of the policy’s effects on the mental health of older adults.

In this study, the panel-ordered probit model was chosen because the dependent variable (mental health score) is an ordered variable ranging from 0 to 30. The ordinal nature of this variable suggests that a standard linear regression model may not be appropriate, as it assumes the dependent variable is continuous and unbounded. The panel-ordered probit model, however, is well-suited to handle this ordinal structure by modeling the probability of the dependent variable falling into specific ordered categories, thereby more accurately reflecting its characteristics. Furthermore, the use of panel data allows for the consideration of unobserved individual-level heterogeneity, which may influence mental health outcomes. By introducing fixed effects, this model reduces the potential bias that could arise from omitted time-invariant variables. Therefore, this model is particularly suitable for analyzing the dynamic effects of the CII policy on elderly mental health. The specific model specifications are as follows:


$${Y_{it}}^{*}=\beta_{\text{0}}+\beta_{\text{1}}M_{it}+{Z^{\prime }}_{it}\delta +u_{i}+\lambda_{t}+\varepsilon_{it}$$ (1)

In this model, $Y_{it}$ represents the observed continuous mental health score, $M_{it}$ is a dummy variable indicating the implementation of the Critical Illness Insurance (CII) policy, and ${Z^{\prime }}_{it}$ is a vector of control variables, including sociodemographic and economic characteristics. The model also incorporates individual fixed effects ($u_{i}$), time fixed effects ($\lambda_{t}$), and a standard normally distributed random error term ($\varepsilon_{it}$). The observed mental health score $Y_{it}$ is derived from a latent variable ${Y_{it}}^{*}$, discretized based on a set of threshold values, as follows:


$$Y_{it}=j\text{当}\tau_{j-\text{1}}<{Y_{it}}^{*}<\tau_{j}$$ (2)

Here, j=0,1,…,30 represents the discrete values of the mental health score, and $\tau_{j}$ denotes the threshold parameters to be estimated. This setup allows the panel-ordered probit model to appropriately model the impact of the Critical Illness Insurance (CII) policy on the ordered response variable.

This study utilizes a panel-ordered probit model combined with a multi-period difference-in-differences (DID) structure to estimate the effect of the CII policy on the mental health of older adults. Specifically, the model aims to estimate the probability of the policy variable $M_{it}$ affecting $Y_{it}$, expressed as:


$$Pr\left(Y_{it}=j\right)=\text{Pr}({\tau_{j-\text{1}}<\beta }_{\text{0}}+\beta_{\text{1}}M_{it}+{Z^{\prime }}_{it}\delta +u_{i}+\lambda_{t}+\varepsilon_{it}<\tau_{j})$$ (3)

Through this modeling framework, the study effectively addresses the characteristics of the ordered dependent variable while incorporating the multi-period DID policy effect across individual and temporal dimensions. This approach aids in identifying the pathways through which the critical illness insurance (CII) policy impacts the mental health of older adults.

In this research, mental health scores were measured using the CES-D Depression Scale, as assessed in the Mental Health section of the CHARLS health questionnaire. The CES-D Depression Scale is a widely recognized tool for evaluating respondents’ psychological well-being and depressive tendencies. Scores on the CES-D range from 0 to 30, with higher scores indicating more severe depressive symptoms. The CES-D scale is commonly employed for screening and assessing depressive symptoms, with researchers and clinicians often setting a threshold to identify individuals at risk of depression. While the threshold can be adjusted according to the study’s objectives and population characteristics, a score of 16 is frequently used as the criterion for diagnosing depression [34–36].

Additionally, some studies adopt a more generalized threshold, considering a score above 10 as indicative of depressive tendencies [37–40]. Given the skewed distribution of CES-D scores, traditional linear regression models may not be suitable. Therefore, this study follows the categorization methods of prior research and adopts a cutoff score of 10 to delineate depressive symptoms. Specifically, respondents with scores greater than 10 are classified as the case group, characterized by “mild or significant depressive symptoms,” while those scoring 10 or below are categorized as the control group, defined as having “no depressive symptoms.” To further ensure robustness, this study also incorporates the 16-point threshold as an additional classification criterion.

### 3.3 Descriptive statistics

Table 1 presents the descriptive statistics of the key variables used in this study. The variables include dependent variables such as depression scores, with the critical illness insurance (CII) policy serving as the primary independent variable. Additionally, the analysis incorporates control variables such as age, gender, marital status, education level, and family size.To further investigate the pathways through which the policy operates, mediating variables such as hospitalization behavior, medical expenditures, catastrophic health expenditures, and family financial support are also included. The descriptive statistics provide the mean and standard deviation for each variable, offering insights into the characteristics of the sample and the distribution of variables. These results establish a solid foundation for the subsequent empirical analysis.

**Table 1:** Descriptive Statistics of Key Variables.

Variable Type	Variable Name	Variable Definition	Mean	Std. Dev.
Dependent Variables	Depression Score	CES-D Depression Score (0–30)	8.600	6.444
	Depressive Tendency (10)	Depression score > 10, 1 = Yes, 0 = No	0.369	0.483
	Depressive Tendency (16)	Depression score > 16, 1 = Yes, 0 = No	0.157	0.364
Independent Variables	Critical Illness Insurance	Affected by policy, 1 = Yes, 0 = No	0.625	0.484
Control Variables	Age	Actual age of respondent (years)	68.621	7.036
	Gender	Female = 1, 0 = Otherwise	0.505	0.500
	Marital Status	Married = 1, 0 = Otherwise	0.783	0.412
	Education Level	Years of education	4.216	4.019
	Disability	Disabled = 1, 0 = Otherwise	0.201	0.401
	Social Activity	Participates = 1, 0 = Otherwise	0.494	0.500
	Household Size	Number of family members	2.995	1.679
	Per Capita Household Income	Log of per capita household income	7.857	1.721
	Health Insurance	Enrolled = 1, 0 = Otherwise	0.933	0.250
	Pension	Enrolled = 1, 0 = Otherwise	0.689	0.463
Mediator Variables	Hospitalization Behavior	Hospitalized = 1, 0 = Otherwise	0.176	0.381
	Number of Hospitalizations	Times hospitalized in the past year	0.281	0.843
	Outpatient Behavior	Outpatient = 1, 0 = Otherwise	0.195	0.396
	Number of Outpatient Visits	Times visited outpatient services in the past year	0.454	1.532
	Total Hospitalization Cost	Total hospitalization cost in the past year (CNY)	2309	13010
	Out-of-Pocket Hospitalization Cost	Out-of-pocket hospitalization cost in the past year (CNY)	1162	6943
	Total Outpatient Cost	Total outpatient cost in the past year (CNY)	293	2578
	Out-of-Pocket Outpatient Cost	Out-of-pocket outpatient cost in the past year (CNY)	181	1797
	Catastrophic Health Expenditure (25%)	Occurred = 1, 0 = Otherwise	0.167	0.373
	Catastrophic Health Expenditure (40%)	Occurred = 1, 0 = Otherwise	0.145	0.352
	Family Financial Support	Amount provided by children in the past year (CNY)	9604	553342
	Family Emotional Support	Number of meetings with children in the past year	15.262	9.825
	Social Support	Types of social activities participated in	1.148	1.012

## 4. Results

### 4.1. Baseline regression results

Columns (1)-(3) of Table 2 present the regression results for the impact of the Critical Illness Insurance (CII) policy on the depression scores of older adults. Column (1) includes only the core explanatory variable (CII), column (2) adds control variables, and column (3) further incorporates time and regional fixed effects to enhance the robustness of the model. The results indicate that the coefficients for the dummy variable representing the CII policy are consistently negative and statistically significant at the 1% level across all model specifications. This demonstrates that the CII policy significantly reduces depression scores among the elderly, thereby effectively improving their mental health. Columns (4)-(5) of Table 2 report the effects of the CII policy on depressive tendencies among older adults, using cut-off scores of 10 and 16, respectively, to define depressive tendencies. The regression coefficients are −0.115 in column (4) and −0.093 in column (5), both statistically significant at the 1% level. These results indicate that regardless of the threshold used to define depressive tendencies, the policy plays a significant role in enhancing the mental health of older adults.

**Table 2:** Results of the Baseline Regression.

	Depression Score			Depressive Tendency	
	(1)	(2)	(3)	(4)	(5)
Critical Illness Insurance	−0.035***	0.014	−0.092***	−0.115***	−0.093*
	(0.012)	(0.015)	(0.028)	(0.039)	(0.049)
Age		−0.006***	−0.006***	−0.006***	−0.009***
		(0.001)	(0.001)	(0.002)	(0.002)
Gender		0.221***	0.240***	0.306***	0.294***
		(0.015)	(0.015)	(0.019)	(0.023)
Marital Status		−0.191***	−0.164***	−0.144***	−0.165***
		(0.019)	(0.019)	(0.024)	(0.028)
Education Level		−0.033***	−0.031***	−0.032***	−0.039***
		(0.002)	(0.002)	(0.003)	(0.003)
Disability		0.335***	0.323***	0.327***	0.374***
		(0.018)	(0.018)	(0.022)	(0.025)
Social Activity		−0.155***	−0.139***	−0.134***	−0.181***
		(0.014)	(0.014)	(0.018)	(0.021)
Household Size		−0.006	−0.015***	−0.016***	−0.028***
		(0.004)	(0.004)	(0.006)	(0.007)
Per Capita Household Income		−0.057***	−0.053***	−0.052***	−0.057***
		(0.004)	(0.004)	(0.006)	(0.007)
Health Insurance		0.006	−0.007	0.018	−0.016
		(0.028)	(0.028)	(0.038)	(0.048)
Pension		−0.006	0.005	−0.004	−0.021
		(0.016)	(0.019)	(0.025)	(0.030)
Time Fixed Effects			YES	YES	YES
Regional Fixed Effects			YES	YES	YES
Number of Observations	29346	21507	22976	22976	22976
Chi-Squared Statistic	8.829	2991.39	2768.87	2073.60	1513.32
P-Value (Prob > χ²)	0.003	0.000	0.000	0.000	0.000
Pseudo Log-Likelihood	−91100	−65400	−69879	−14035	−9098

Note: Values in parentheses represent standard errors. *, **, and *** denote significance levels of 10%, 5%, and 1%, respectively (same as below).

Further analysis reveals that the estimated effects of control variables align with expectations. Age is significantly and negatively correlated with depression scores, while higher levels of education, marital support, and better economic conditions are all associated with a reduced risk of depression. Additionally, social engagement exhibits a significant negative effect on depression scores, suggesting that older adults who participate in social activities experience fewer depressive symptoms. Robustness checks using alternative model specifications and depressive tendency thresholds yield consistent results, further substantiating the positive impact of the CII policy.

In summary, the findings from the panel-ordered probit model demonstrate that the CII policy significantly reduces depression scores and depressive tendencies among older adults. These results are robust across various model specifications and definitions of depression, underscoring the policy’s critical role in improving the mental health of older adults. The study highlights the policy’s significant value in mitigating depressive symptoms and advancing mental health outcomes in the aging population.

### 4.2. Parallel trends test

Before employing the gradual difference-in-differences (DID) model, verifying the parallel trends assumption is a critical prerequisite. This assumption requires that, prior to policy implementation, the trends in mental health changes for the treatment and control groups are consistent. This ensures that any observed effects on mental health can be attributed to the Critical Illness Insurance (CII) policy rather than external factors. This study adopts the event study method proposed by Jacobson et al. (1993), constructing relative time variables to capture the dynamic effects before and after policy implementation [41]. This approach tests whether the parallel trends assumption holds, validating the causal identification of the policy effect. The model is specified as follows:


$$\begin{array}{c} Y_{it}=\beta_{\text{0}}+\sum {\mathrm{\backslash nolimits}}_{t=-\text{4}}^{\text{4}}\delta_{t}D_{it}+\beta_{\text{4}}X_{it}+\eta_{i}+\gamma_{t}+\varepsilon_{it} \end{array}$$ (4)

In this model, $Y_{it}$represents the mental health score of individual i at time t; $D_{it}$s a dummy variable for the relative timing of policy implementation, taking a value of 1 if the Critical Illness Insurance (CII) policy was implemented in year t in a given region, and 0 otherwise. Control variables $X_{it}$ include sociodemographic characteristics and other factors potentially influencing mental health. Individual fixed effects $\eta_{i}$nd time fixed effects $\gamma_{t}$ are included to account for unobserved heterogeneity. The key coefficient $\delta_{t}$ measures the difference in mental health scores between pilot and non-pilot regions in year t relative to policy implementation.

**Fig 1:**
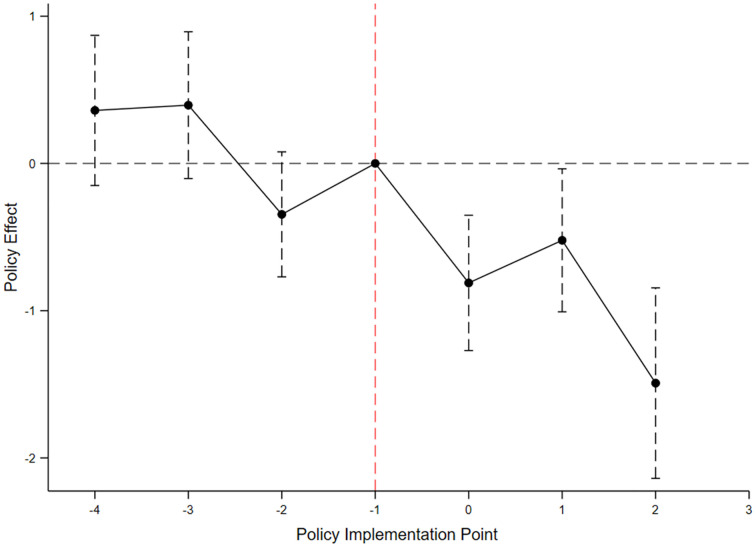
Parallel trends test of the CII policy’s effect on elderly mental health. Notes: Estimated coefficients with 95% confidence intervals show no significant differences before policy implementation, supporting the DID assumption.

As shown in the parallel trends test results (see Fig 1), the coefficients δt for all pre-policy periods are statistically insignificant. This indicates no significant difference in mental health trends between the treatment and control groups before policy implementation, thereby satisfying the parallel trends assumption.

These findings provide strong support for the use of the multi-period DID model, allowing the identification of the CII policy’s impact on mental health as the net effect of the policy intervention.

### 4.3. Robustness test

#### 4.3.1. Placebo test.

To evaluate the causal effect of the Critical Illness Insurance (CII) policy and verify the robustness of the model, this study conducted a placebo test following the methods of La Ferrara et al. (2012) and Li et al. (2022) [42, 43]. Specifically, in cities where the policy was not implemented, a “policy implementation year” was randomly assigned to create a placebo treatment variable (placebo_treat). Based on this variable, 500 regressions were performed, recording the estimated coefficients, standard errors, and p-values.

**Fig 2:**
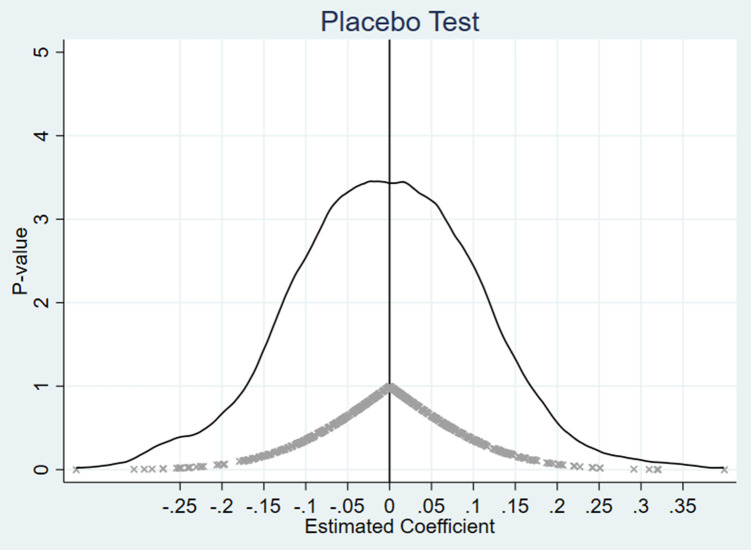
Placebo test of the CII policy’s effect on elderly mental health. Notes: Estimated coefficients with 95% confidence intervals indicate no significant effects under placebo assignments, confirming robustness.

The distribution of the estimated coefficients was visualized (see Fig 2) to examine whether the placebo treatment variable’s estimates were concentrated around zero, thereby assessing the potential for omitted variable bias. The results indicate that most of the placebo treatment coefficients are clustered near zero, follow a normal distribution, and are statistically insignificant. This confirms that the randomly assigned placebo policy timing did not produce significant effects, validating the robustness of the model.

In summary, the placebo test results further support the robustness of the research model, confirming the causal effect of the CII policy on mental health. These findings ensure that the estimated impact of the CII policy reflects genuine policy effects rather than model specification issues or interference from exogenous factors.

#### 4.3.2. Robustness test with alternative econometric models.

To further validate the robustness of the Critical Illness Insurance (CII) policy’s impact on the mental health of older adults, this study employs multiple econometric methods, including ordinary least squares (OLS), high-dimensional fixed effects (HDFE) models, panel fixed effects (Panel FE) models, panel random effects (Panel RE) models, and ordered logit models. The empirical results indicate that in most model specifications, the estimated coefficients of the policy effect on mental health are negative and statistically significant. Specifically, the OLS, HDFE, and Panel RE models show negative policy effect coefficients significant at the 5% level, demonstrating that the policy significantly reduces depression scores and has a positive impact on alleviating mental health issues among older adults. The ordered logit model also yields statistically significant results, further supporting the negative impact of the policy on depression, as shown in Table 3.

**Table 3:** Robustness Estimates Using Alternative Econometric Models.

	OLS	HDFE	Panel FE	Panel RE	Ordered Logit
	(1)	(2)	(3)	(4)	(5)
Critical Illness Insurance	−0.367**	−0.367**	−0.139	−0.367**	−0.110**
	(0.167)	(0.167)	(0.297)	(0.166)	(0.050)
Control Variable	YES	YES	YES	YES	YES
Time Fixed Effects	YES	YES	YES	YES	YES
Regional Fixed Effects	YES	YES	YES	YES	YES
Number of Observations	21507	21507	21507	21507	21507
Chi-Squared Statistic	0.163	0.163	0.1602	0.1514	——
P-Value (Prob > χ²)	96.80	194.79	31.94	4372.23	3195.89
Pseudo Log-Likelihood	0.000	0.000	0.000	0.000	0.000
Time Fixed Effects	——	——	——	——	−6.51E + 04

The robustness analysis confirms that the policy effect remains consistent across various model specifications. Although the Panel FE model results do not reach traditional significance thresholds, the direction of the estimates aligns with those of other models, indicating no substantial deviation. All models produce p-values below 0.05, providing additional evidence for the statistical significance of the findings.

#### 4.3.3. PSM-DID estimation.

This study employs a propensity score matching combined with difference-in-differences (PSM-DID) model to estimate the causal effect of the Critical Illness Insurance (CII) policy on the mental health of older adults. Given the non-random nature of policy implementation, selection bias may arise due to differences in healthcare resources and economic conditions between pilot and non-pilot regions. The PSM method is applied to construct comparable treatment and control groups, thereby reducing selection bias and enhancing the reliability of policy effect estimation.

After matching, the DID method is used to evaluate the policy’s impact, dynamically capturing its temporal effects while controlling for observed confounding factors. In the empirical analysis, three matching methods—nearest-neighbor matching, caliper matching, and kernel matching—are employed. All matching results consistently show a policy coefficient of −0.4618, significant at the 1% level, indicating that the policy significantly reduces depressive symptoms and improves mental health among older adults. The consistency across the three methods further confirms the robustness and credibility of the findings, as shown in Table 4.

**Table 4:** Robustness Estimates of PSM-DID.

	Nearest Neighbor Matching	Caliper Matching	Kernel Matching
	(1)	(2)	(3)
Critical Illness Insurance	‘-0.4618***	’-0.4602***	‘-0.4618***
	(0.1644)	(0.1643)	(0.1644)
Control Variable	YES	YES	YES
Time Fixed Effects	YES	YES	YES
Regional Fixed Effects	YES	YES	YES
Number of Observations	22961	22975	22961
Adj R-squared	0.129	0.129	0.129

### 4.4. Mechanism analysis

#### 4.4.1. Healthcare utilization pathway.

This study systematically examined the mediating roles of inpatient and outpatient service utilization between the Critical Illness Insurance (CII) policy and mental health outcomes in older adults using stepwise regression and the Sobel test. The results show that the CII policy significantly increased the utilization of inpatient services (with both hospitalization probability and frequency rising), yet inpatient treatment was associated with a worsening of depression (with a significantly positive coefficient). This may be due to hospitalization often being linked with the diagnosis of severe illnesses, treatment-related suffering, and health concerns, and the increased frequency of hospitalizations may reflect more serious health issues. Meanwhile, the policy significantly reduced outpatient service utilization (with both outpatient visits and frequency decreasing), but outpatient use was similarly associated with a worsening of depression, as shown in Table 5.

**Table 5:** Mediation Effect Test of Healthcare Service Utilization Pathways.

	Hospitalized	Depression Score	Number of Hospitalizations	Depression Score	Outpatient	Depression Score	Number of Outpatient Visits	Depression Score
	(1)	(2)	(3)	(4)	(5)	(6)	(7)	(8)
Critical Illness Insurance	0.042***	−0.557***	0.089***	−0.554***	−0.024***	−0.524***	−0.096***	−0.558***
	(0.005)	(0.164)	(0.011)	(0.164)	(0.006)	(0.163)	(0.022)	(0.164)
Hospitalized		1.850***						
		(0.114)						
Number of Hospitalizations				0.847***				
				(0.072)				
Outpatient						1.931***		
						(0.104)		
Number of Outpatient Visits								0.368***
								(0.037)
Control Variable	YES	YES	YES	YES	YES	YES	YES	YES
Number of Observations	26593	22971	26583	22965	26582	22963	26371	22797
R²	0.014	0.137	0.011	0.136	0.007	0.14	0.004	0.132
Sobel Z-Value	8.039		6.347		−3.679		−3.710	
Sobel Z-p Value	0.0000		0.0000		0.0002		0.0002	

This suggests that, after alleviating the economic burden of healthcare, the CII policy may have altered the healthcare-seeking patterns of older adults: on the one hand, it encourages seriously ill patients to opt for inpatient care, while on the other hand, the reduction in outpatient services may mean that less severe patients failed to receive timely interventions. The Sobel test confirmed that the mediating effects of both types of services were significant, highlighting that changes in the utilization of medical services are an important pathway through which the policy impacts mental health.

#### 4.4.2. Medical cost-sharing pathway.

This study reveals the economic burden mechanism through both inpatient and outpatient expenditure pathways, illustrating how the Critical Illness Insurance (CII) policy affects the mental health of older adults, as shown in Table 6. In terms of inpatient expenditure, the policy significantly increased both total inpatient expenditure and out-of-pocket expenses, leading to persistent medical financial pressure, thereby causing psychological burden for older adults. The Sobel test confirmed the significant mediating effect of total inpatient expenditure.

**Table 6:** Mediation Effect Test of Healthcare Expenditure Pathways.

	Total Hospitalization Cost	Depression Score	Out-of-Pocket Hospitalization Cost	Depression Score	Total Outpatient Cost	Depression Score
	(1)	(2)	(3)	(4)	(5)	(6)
Critical Illness Insurance	1228.5***	−0.551***	607.4***	−0.551***	72.469**	−0.548***
	(157.5)	(0.165)	(80.88)	(0.165)	(32.79)	(0.166)
Total Hosp. Cost		0.00002***				
		0.000				
OOP Hosp. Cost				0.00005***		
				0.000		
Total Outpt. Cost						0.00016***
						0.000
Control Variable	YES	YES	YES	YES	YES	YES
Number of Observations	26222	22708	26226	22708	26204	22644
R²	0.009	0.129	0.006	0.128	0.002	0.127
Sobel Z-Value	4.589		4.457		1.997	
Sobel Z-p Value	0.000		0.000		0.046	

The outpatient expenditure pathway presents more complex characteristics: although the policy slightly increased total outpatient expenditure, it led to a larger increase in out-of-pocket expenses, reflecting a structural gap in the compensation mechanism. Notably, both types of outpatient expenditure were significantly positively correlated with depression levels, and the influencing mechanism may involve dual pressures: one is the economic burden of absolu te medical expenditure, and the other is the signal of deteriorating health implied by the increased expenditure.

#### 4.4.3. Healthcare reimbursement pathways.

This study reveals the mechanism through which the Critical Illness Insurance (CII) policy affects the mental health of older adults via differentiated healthcare reimbursement pathways, as shown in Table 7. Empirical analysis shows that the policy significantly increased the reimbursement rates for both inpatient and outpatient services, but the impacts of these two reimbursement pathways on mental health differ significantly. Specifically, an increase in the inpatient reimbursement rate has a significant negative effect on depression scores, confirming that improving reimbursement for inpatient costs effectively alleviates the psychological stress of older adults. In contrast, although the outpatient reimbursement rate also increased, its mediating effect did not reach statistical significance, reflecting the limited role of the outpatient reimbursement mechanism in improving mental health.

**Table 7:** Mediation Effect Test of Health Insurance Reimbursement Pathways.

	Hospitalization Reimbursement Ratio	Depression Score	Outpatient Reimbursement Ratio	Depression Score
	(1)	(2)	(3)	(4)
Critical Illness Insurance	4.988***	−1.314***	2.044**	−0.526
	(1.091)	(0.446)	(0.874)	(0.377)
Hospitalization Reimbursement Ratio		−0.012***		
		(0.004)		
Outpatient Reimbursement Ratio				−0.002
				(0.003)
Control Variable	YES	YES	YES	YES
Number of Observations	4396	3682	5461	4762
R²	0.048	0.149	0.035	0.151
Sobel Z-Value	−2.348		−1.521	
Sobel Z-p Value	0.019		0.128	

This difference may stem from the following factors: (1) inpatient costs are generally higher, and the economic relief effect from increased reimbursement is more pronounced; (2) inpatient patients are often more severely ill, and cost compensation has a stronger psychological soothing effect on them; (3) the increase in outpatient reimbursement is relatively small, making it difficult to produce substantial psychological effects. The findings suggest that in improving the healthcare security system, more attention should be given to the inpatient reimbursement mechanism, while also optimizing the design of outpatient reimbursement policies to enhance their mental health promotion effects.

#### 4.4.4. Financial risk pathway.

This study investigates the impact of the Critical Illness Insurance (CII) policy on the mental health of older adults through the financial risk prevention and control pathway. Specifically, the analysis uses catastrophic health expenditure (CHE) thresholds of 25% and 40% of household income as mediators, with the 40% threshold being widely recognized in the literature [44, 45].

The results show that the policy significantly reduces the probability of catastrophic health expenditures at the 25% threshold, which in turn significantly lowers depression scores, as shown in Table 8. This suggests that the policy is more effective in alleviating moderate financial burdens. At the 40% threshold, the impact coefficient is −0.118, with a mediating effect accounting for 11.82%. Although the policy’s impact on high financial risk is weaker at this threshold, it remains statistically significant, demonstrating that the policy continues to have a positive effect on reducing the psychological stress associated with high medical costs.

**Table 8:** Mediation Effect Test of Financial Risk Pathways.

	Catastrophic Health Expenditure (25%)	Depression Score	Catastrophic Health Expenditure (40%)	Depression Score
	(1)	(2)	(3)	(4)
Critical Illness Insurance	−0.027*	−0.530***	−0.118**	−0.075**
	(0.015)	(0.163)	(0.055)	(0.036)
Catastrophic Health Expenditure (25%)		1.998***		
		(0.118)		
Catastrophic Health Expenditure (40%)				0.288***
				(0.023)
Control Variable	YES	YES	YES	YES
Number of Observations	26602	22976	20955	17840
R²	0.057	0.1311	0.0719	0.0311
Sobel Z-Value	4.662		3.888	
Sobel Z-p Value	0.0000		0.0001	

When comparing the two thresholds, it is evident that the mediating effect is significantly stronger at the 25% threshold than at the 40% threshold. This highlights that the CII policy is more effective at mitigating the psychological impact of moderate financial risk but less impactful in addressing high expenditure burdens. Overall, by reducing the probability of catastrophic health expenditures, the CII policy plays a crucial role in alleviating the financial and mental health pressures associated with healthcare expenses, particularly for those facing low to moderate financial risk.

#### 4.4.5. Social support pathway.

This study systematically examines the impact of the Critical Illness Insurance (CII) policy on the mental health of older adults through the social support pathway, as shown in Table 9. The analysis shows that the policy significantly increased the level of economic support older adults received from their families, and this enhanced economic support effectively alleviated their depressive symptoms. At the same time, the study found that after the policy implementation, the frequency of intergenerational interactions between older adults and their children decreased, which had a negative impact on their mental health. Additionally, the policy significantly increased the frequency of social participation among older adults, and this enhanced social support had a positive effect on improving their mental health.

**Table 9:** Mediation Effect Test of Social Support Mechanisms.

	Family Financial Support	Depression Score	Family Emotional Support	Depression Score	Social Support	Depression Score
	(1)	(2)	(3)	(4)	(5)	(6)
Critical Illness Insurance	1064.412***	−0.485***	−1.729***	−0.449***	0.084***	−0.699***
	(193.5)	(0.167)	(0.132)	(0.170)	(0.016)	(0.208)
Family Financial Support		−0.000***				
		(0.000)				
Family Emotional Support				−0.028***		
				(0.005)		
Social Support						−0.482***
						(0.056)
Control Variable	YES	YES	YES	YES	YES	YES
Number of Observations	23179	19824	24688	21287	17763	15114
R²	0.016	0.122	0.15	0.127	0.478	0.132
Sobel Z-Value	−3.634		6.756		−4.881	
Sobel Z-p Value	0.000		0.000		0.000	

Specifically, the CII policy impacts the social support system of older adults through three dimensions: in terms of family economic support, the policy alleviated the medical expenditure burden, encouraging children to increase financial support for their parents; in terms of intergenerational relationships, the substitution effect of economic support led to a reduction in face-to-face emotional communication; in terms of social interactions, the reduced medical burden created more conditions and opportunities for older adults to participate in social activities. The results show that although the decrease in intergenerational interactions brought some negative effects, the enhancement of family economic support and social support overall had a positive effect on the mental health of older adults. These findings provide important insights for improving healthcare security policies.

### 4.5. Heterogeneity analysis

#### 4.5.1. Income heterogeneity analysis.

The income heterogeneity analysis highlights significant differences in the mental health impact of the Critical Illness Insurance (CII) policy across income groups, as shown in Table 10. The results of the income heterogeneity analysis indicate that the Critical Illness Insurance (CII) policy significantly improves the depression scores of middle-income and high-income groups, while having no statistically significant impact on the mental health of the low-income group. Specifically, the policy coefficient for the depression score of the low-income group is −0.318, which does not reach statistical significance, suggesting that the policy has a limited effect on improving mental health for this population. In contrast, the policy coefficients for the middle-income and high-income groups are both statistically significant at the 5% level, indicating substantial improvements in the mental health of these two groups.

**Table 10:** Empirical Results of Heterogeneity by Income Groups.

	Low-Income	Middle-Income	High-Income
	(1)	(2)	(3)
Critical Illness Insurance	−0.318	−0.664**	−0.521**
	(0.292)	(0.322)	(0.250)
Control Variable	YES	YES	YES
Time Fixed Effects	YES	YES	YES
Regional Fixed Effects	YES	YES	YES
Number of Observations	7568	7588	7820
R²	0.099	0.111	0.107

Further analysis reveals that this heterogeneity reflects the “ground conditions effect.” For middle- and high-income groups, their relatively stable economic foundations enable the CII policy to effectively alleviate the burden of medical expenses, leading to significant improvements in mental health. Conversely, the low-income group faces greater financial stress, and the medical support provided by the policy is insufficient to fully alleviate their broader economic pressures. This limitation diminishes the policy’s capacity to significantly enhance mental health outcomes for low-income individuals.

#### 4.5.2. Health Status Heterogeneity Analysis.

The health status heterogeneity analysis underscores that the mental health benefits of the Critical Illness Insurance (CII) policy vary significantly depending on the physical health conditions of elderly individuals, as shown in Table 11. The analysis of health status heterogeneity reveals significant differences in the impact of the Critical Illness Insurance (CII) policy on the mental health of the elderly, depending on their health conditions. For older adults without chronic diseases, the policy coefficient for depression scores is −0.218 and does not reach statistical significance, indicating that the policy has no notable effect on the mental health of this group. In contrast, for those with chronic diseases, the policy coefficient is −0.485 (significant at the 5% level), while the group with multiple co-morbidities exhibits significant improvements at the 10% significance level. These findings suggest that the policy has a substantial positive impact on the mental health of individuals in these latter two groups.

**Table 11:** Empirical Results of Heterogeneity by Health Groups.

	No Chronic Disease	With Chronic Disease	Multiple Chronic Conditions
	(1)	(2)	(3)
Critical Illness Insurance	−0.218	−0.485**	−0.497*
	(0.310)	(0.246)	(0.258)
Control Variable	YES	YES	YES
Time Fixed Effects	YES	YES	YES
Regional Fixed Effects	YES	YES	YES
Number of Observations	6430	10039	8624
R²	0.109	0.147	0.143

#### 4.5.3. Regional heterogeneity analysis.

The regional heterogeneity analysis demonstrates that the mental health effects of the Critical Illness Insurance (CII) policy differ markedly across geographic regions in China, as shown in Table 12. The analysis of regional heterogeneity reveals that the impact of the Critical Illness Insurance (CII) policy on mental health varies significantly across regions. In the eastern region, the policy coefficient for depression scores is 0.033 and does not reach statistical significance, indicating that the policy has no notable effect on mental health in this area. Conversely, in the central region, the policy coefficient is −0.822 (significant at the 1% level), demonstrating that the policy significantly reduces depression scores and improves mental health. Similarly, a significant improvement in mental health is observed in the western region.

**Table 12:** Empirical Results of Heterogeneity by Regional Groups.

	Eastern Region	Central Region	Western Region
	(1)	(2)	(3)
Critical Illness Insurance	0.033	−0.822***	−0.667**
	(0.300)	(0.278)	(0.319)
Control Variable	YES	YES	YES
Time Fixed Effects	YES	YES	YES
Regional Fixed Effects	YES	YES	YES
Number of Observations	6318	7747	7772
R²	0.077	0.106	0.116

These results highlight the influence of regional disparities in economic development and the allocation of social security resources on the effectiveness of the policy. In the central and western regions, where economic foundations are weaker and social security resources are less abundant, the policy is more effective in alleviating the burden of medical expenses and enhancing mental health. In contrast, in the eastern region, higher economic levels and a more developed social security system have largely addressed the population’s healthcare needs. Consequently, the marginal effect of the policy in improving mental health is smaller and statistically insignificant.

## 5. Discussion

This study assesses the impact of the Critical Illness Insurance (CII) policy on the mental health of older adults using a multi-period difference-in-differences (DID) approach. The CII policy was implemented at different times across regions in China, providing a “quasi-natural experiment” setup. The findings support previous studies while adding new insights into the mechanisms at play and variations across different groups.

First, the study shows that the CII policy significantly reduces depression and improves mental health among older adults. This aligns with earlier research on the positive effects of health insurance on mitigating health risks [5, 7]. The effects are particularly strong among middle- and high-income individuals, those with chronic illnesses, and older adults in central and western China. These findings highlight the policy’s role in addressing health disparities.

Second, we identified several pathways through which the CII policy affects mental health, including increased healthcare utilization, cost-sharing, financial risk reduction, and social support [27, 28]. The main driver of improved mental health is the increased use of inpatient services, which is consistent with health needs theory. By covering more hospitalization costs, the policy reduces the financial burden of illness, helping to prevent poverty and financial setbacks. However, the policy’s effect on outpatient services and emotional support suggests there may be unintended consequences, such as reduced reliance on family support. This “institutional substitution” effect warrants further exploration [44].

While these findings are robust, the study does have limitations. First, mental health is mainly measured by depression scores, which may not capture all aspects of mental well-being. Second, we have not fully explored how the policy impacts different subgroups within the elderly population, such as age groups or urban versus rural areas. Finally, the study’s short time span limits our ability to assess long-term effects of the policy, which could provide a more complete picture of its impact.

Based on these findings, we propose the following policy recommendations:

1. Strengthen the precise coverage of the CII policy: The CII policy should further strengthen coverage for low-income groups and economically underdeveloped regions, ensuring that these populations can equitably benefit from the health improvements brought by the policy. This will enhance the precision and fairness of the policy.
2. Focus on inpatient cost coverage in the CII policy design: The policy should continue to focus on covering inpatient expenses while gradually improving outpatient service coverage. By increasing reimbursement rates for hospitalization, the policy can alleviate the economic burden on older adults and further enhance its health improvement effects.
3. Strengthen the prevention and control of catastrophic health expenditures: The policy should increase its capacity to prevent and control catastrophic health expenditures, further reducing the financial shock that medical costs bring to households, particularly in cases of major illness. Increasing the reimbursement limits for catastrophic health expenditures will help prevent households from falling into poverty or financial distress due to high medical costs.

## 6. Conclusion

This study systematically assessed the impact of the Critical Illness Insurance (CII) policy on the mental health of older adults. The key findings are as follows: First, the CII policy significantly reduced depression scores and improved overall mental health among older adults. The policy was particularly effective among middle- and high-income groups, individuals with chronic illnesses, and elderly populations in central and western China. Mechanistic analysis revealed that the CII policy improved mental health through four primary pathways: increased utilization of medical services, medical cost-sharing, financial risk mitigation, and enhanced social support. Among these, the increase in inpatient service utilization emerged as the core driver of the policy’s effectiveness. By raising reimbursement rates for hospitalization costs, the policy significantly reduced the risks of poverty and financial distress due to illness.However, some unintended effects, such as the substitution effect of outpatient services and the weakening of family emotional support, indicate that the policy design needs to balance potential trade-offs while expanding its scope.

## Supporting information

S1 File Human subjects research checklist. (DOCX)
